# Cirrhosis with COVID‐19: Just another infection or something more to it?

**DOI:** 10.1002/jgh3.13031

**Published:** 2023-12-30

**Authors:** Dibyalochan Praharaj, Madhumita Premkumar

**Affiliations:** ^1^ Department of Gastroenterology and Hepatology Kalinga Institute of Medical Sciences and Pradyumna Bal Memorial (PBM) hospital Bhubaneswar India; ^2^ Department of Hepatology Postgraduate Institute of Medical Education and Research Chandigarh India

In this issue of the *JGH Open*, Sohail *et al.* describe the predictors of inpatient outcomes of COVID‐19 infection in patients with cirrhosis in the early pandemic phase. This retrospective cohort study encompasses two clinical databases wherein the authors look for the early clinical predictors of clinical outcome in patients with cirrhosis and COVID‐19. It is a fairly large cohort of 73 354 cirrhosis‐related hospitalizations from North America, of whom 25 194 had concomitant cirrhosis and COVID‐19 infection. The study specifically looked into the effect of COVID‐19 in patients with compensated or decompensated cirrhosis (DC). Moreover, the study describes the readmission rate and causes of hospital readmission in cirrhotic patients with concomitant COVID‐19. Subgroup analysis showed that patients with DC tend to have increased mortality, longer hospitalization and increased cost of hospitalization. Similarly, on multivariate analysis, the presence of COVID‐19 infection, age, Charlson's co‐morbidity index >3, acute kidney injury, end‐stage renal disease, septic shock, acute respiratory failure, mechanical ventilation, and ICU status were found to be the independent predictors of mortality.^1^

Patients with cirrhosis often have an acquired immunodeficiency, often labeled as cirrhosis‐associated immune dysfunction (CAID), which makes these individuals susceptible to various bacterial, viral, and fungal infections. Spontaneous bacterial peritonitis, urinary tract infection, pneumonia, and soft tissue infections are common in patients with DC.^2^ In addition to CAID, there is significant alteration in gut mucosal barrier, which leads to increased translocation of various bacteria and bacterial products (endotoxins). The final result is cytokine storm and multi‐organ dysfunction.^3^

The current ongoing COVID‐19 pandemic has posed significant challenge to healthcare systems worldwide and is associated with increased morbidity and mortality. The COVID‐19 infection in a patient with underlying cirrhosis may per se lead to worsening liver function or even acute‐on‐chronic liver failure (ACLF). Mortality in patients with cirrhosis may be higher in comparison with their non‐cirrhotic counterparts due to underlying immune dysfunction, malnutrition, and various underlying comorbidities.^4^ In fact, recipients were shown to have difficult post‐liver transplantation (LT) outcomes.^5^

The most common hepatic manifestation of COVID‐19 infection includes elevated transaminases. Elevated serum bilirubin is rare. Some patients have low serum albumin levels. Patients with severe COVID‐19 infection more commonly have derangement in liver enzymes.^6^

In a registry‐based study by Majrot T *et al.*, 745 patients with COVID‐19 infection and CLD were included (386 patients with cirrhosis and 359 patients without cirrhosis). The clinical outcome of these patients was compared with COVID‐19 positive patients without CLD from a UK hospital network. Mortality was higher in patients with cirrhosis (32%) than those without (8%). Although the most common cause of death was extrahepatic, that is, related to respiratory failure, the rate of mortality progressively increased with increase in Child–Turcotte–Pugh score. More importantly, acute hepatic decompensation developed in about 46% patients with cirrhosis, of which 21% of patients surprisingly had no respiratory symptoms.^7^ Half of the patients with acute decompensation developed ACLF.^7^

Few other studies have also noted poor outcome in patients with cirrhosis who have COVID‐19 infection.^4^, ^8^, ^9^ A multicenter study from the United States by Bajaj JS *et al.* compared mortality in patients of cirrhosis with COVID‐19 infection against patients with isolated cirrhosis/Covid‐19 infection. They found out that patients with cirrhosis and COVID‐19 had higher mortality as compared with COVID‐19 alone. In contrast, mortality rate was similar in patients with cirrhosis and cirrhosis + COVID‐19 infection. Another interesting observation was in patients who developed ACLF (NACSELD‐ACLF) mortality was similar irrespective of presence or absence of COVID‐19 infection. Thus, it can be concluded that the severity of underlying liver disease is the ultimate killer rather than the COVID‐19 infection. However, inferences should be drawn very carefully from this study because of the lower number of patients enrolled in the cirrhosis + COVID‐19 arm, which might have led to an unbalanced sample size comparison.^9^

In contrast, a multicentric European study by Brozat JF *et al.* showed that mortality in patients with COVID‐19 infection and cirrhosis is primarily determined by other comorbidities, severity of liver, and multi‐organ failure. After propensity score matching case, fatality rate was similar in patients with or without cirrhosis.^10^

The outline and important results of all these studies are shown in Table 1.^1^, ^4^, ^7^, ^9^, ^10^

**Table 1:** Outline of studies describing the outcome of COVID‐19 infection in patients with cirrhosis

Study details/year/author	Nature of study	Primary objective and outcomes	Number of patients with cirrhosis	Results of the study
Index study (Sohail et al., 2023)^1^	Retrospective record based cohort study from United States	In patient mortality/MV/ICU stay and LOS	25 194 patients with cirrhosis and COVID‐19	Patients with DC had higher mortality, longer hospital stay and higher hospitalization cost
Sarin SK et al. (2020)^4^	Multicenter web based Performa based retrospective study from Asia	Spectrum of liver injury, complications of liver disease and COVID‐19 related complications in relation to pre‐existing CLD with or without cirrhosis and its spectrum with SARS CoV2 infection	27 patients with cirrhosis and COVID‐19 infection	Acute liver injury more common, progressive and severe in decompensated cirrhosis 20% patients with cirrhosis had ACLF or AD
Marjot et al. (2021)^7^	International registry‐based cohort study	To determine the impact of COVID‐19 infection in patients with underlying chronic liver disease	386 patients with cirrhosis and COVID‐19	With progressive worsening of cirrhosis there is significant increase in mortality
Bajaj et al. (2020)^9^	Multicenter retrospective study from US	Development of ACLF/Acute decompensation/Mortality in patients with cirrhosis	37 patients with cirrhosis + COVID‐19;108 patients with COVID‐19 and 127 patients with cirrhosis only	Patients with cirrhosis + COVID‐19 have higher mortality compared with COVID‐19 without cirrhosis
Brozat et al. (2022)^10^	Multicenter hospital record based cohort study	To describe group differences in symptoms, inflammatory parameters, liver functions, and organ failure between patients with cirrhosis and matched controls without cirrhosis.	70 patients with cirrhosis and COVID‐19 infection	Patients with cirrhosis show fewer symptoms as compared to patients without cirrhosis. Mortality in patients with cirrhosis similar to without cirrhosis after PSM

ACLF, acute‐on‐chronic liver failure; DC, decompensated cirrhosis; ICU, intensive care unit; LOS, length of stay; MV, mechanical ventilation; PSM, propensity score matching.

However, given the retrospective nature of these data, and the differential effect of COVID‐19 related healthcare disruptions affected ICU admissions and routine care, these COVID‐19 related database studies are limited by lack of homogeneity. Many of the patients with cirrhosis failed to receive routine care and those with worse liver disease severity may have been at higher risk of secondary organ failures and mortality. Similarly, the study by Sohail *et al.* did not have information regarding MELD‐Na or LT. Theoretically, mortality may be higher in patients with cirrhosis and COVID‐19 in view of underlying CAID, which may be even more prominent in patients with DC due to gut dysbiosis and bacterial translocation. The lessons from the pandemic may be to ensure equitable access to vaccines, provision to routine healthcare, and access to LT.
